# A region-based gene association study combined with a leave-one-out sensitivity analysis identifies *SMG1* as a pancreatic cancer susceptibility gene

**DOI:** 10.1371/journal.pgen.1008344

**Published:** 2019-08-30

**Authors:** Cavin Wong, Fei Chen, Najmeh Alirezaie, Yifan Wang, Adeline Cuggia, Ayelet Borgida, Spring Holter, Tatiana Lenko, Celine Domecq, Alzheimer’s Disease Neuroimaging Initiative, Gloria M. Petersen, Sapna Syngal, Randall Brand, Anil K. Rustgi, Michele L. Cote, Elena Stoffel, Sara H. Olson, Nicholas J. Roberts, Mohammad R. Akbari, Jacek Majewski, Alison P. Klein, Celia M. T. Greenwood, Steven Gallinger, George Zogopoulos

**Affiliations:** 1 The Research Institute of the McGill University Health Centre, Montreal, Quebec, Canada; 2 The Goodman Cancer Research Centre of McGill University, Montreal, Quebec, Canada; 3 Johns Hopkins Bloomberg School of Public Health, Baltimore, Maryland, United States of America; 4 McGill University and Genome Quebec Innovation Centre, Montreal, Quebec, Canada; 5 Lunenfeld-Tanenbaum Research Institute, Mount Sinai Hospital, Toronto, Ontario, Canada; 6 Department of Health Sciences Research, Mayo Clinic, Rochester, Minnesota, United States of America; 7 Division of Cancer Genetics and Prevention, Dana-Farber Cancer Institute, Gastroenterology Division, Brigham and Women’s Hospital, Harvard Medical Schozol, Boston, Massachusetts, United States of America; 8 Department of Medicine, University of Pittsburgh, Pittsburgh, Pennsylvania, United States of America; 9 Division of Gastroenterology, Departments of Medicine and Genetics, Pancreatic Cancer Translation Center of Excellence, Abramson Cancer Center, University of Pennsylvania Perelman School of Medicine, Philadelphia, Pennsylvania, United States of America; 10 Karmanos Cancer Institute, Wayne State University School of Medicine, Detroit, Michigan, United States of America; 11 Department of Internal Medicine, University of Michigan, Ann Arbor, Michigan, United States of America; 12 Department of Epidemiology and Biostatistics, Memorial Sloan Kettering Cancer Center, New York, New York, United States of America; 13 Sidney Kimmel Comprehensive Cancer Center at Johns Hopkins, Baltimore, Maryland, United States of America; 14 The Sol Goldman Pancreatic Cancer Research Center, Department of Pathology, Johns Hopkins University, Baltimore, Maryland, United States of America; 15 Women’s College Hospital Research Institute, Women's College Hospital, Toronto, Ontario, Canada; 16 Dalla Lana School of Public Health, University of Toronto, Toronto, Ontario, Canada; 17 Ludmer Centre for Neuroinformatics & Mental Health, McGill University, Montreal, Quebec, Canada; 18 Lady Davis Institute, Jewish General Hospital, McGill University, Montreal, Quebec, Canada; 19 Gerald Bronfman Department of Oncology, and Department of Epidemiology, Biostatistics and Occupational Health, McGill University, Montreal, Quebec, Canada; Cleveland Clinic Genomic Medicine Institute, UNITED STATES

## Abstract

Pancreatic adenocarcinoma (PC) is a lethal malignancy that is familial or associated with genetic syndromes in 10% of cases. Gene-based surveillance strategies for at-risk individuals may improve clinical outcomes. However, familial PC (FPC) is plagued by genetic heterogeneity and the genetic basis for the majority of FPC remains elusive, hampering the development of gene-based surveillance programs. The study was powered to identify genes with a cumulative pathogenic variant prevalence of at least 3%, which includes the most prevalent PC susceptibility gene, *BRCA2*. Since the majority of known PC susceptibility genes are involved in DNA repair, we focused on genes implicated in these pathways. We performed a region-based association study using the Mixed-Effects Score Test, followed by leave-one-out characterization of PC-associated gene regions and variants to identify the genes and variants driving risk associations. We evaluated 398 cases from two case series and 987 controls without a personal history of cancer. The first case series consisted of 109 patients with either FPC (n = 101) or PC at ≤50 years of age (n = 8). The second case series was composed of 289 unselected PC cases. We validated this discovery strategy by identifying known pathogenic *BRCA2* variants, and also identified *SMG1*, encoding a serine/threonine protein kinase, to be significantly associated with PC following correction for multiple testing (p = 3.22x10^-7^). The *SMG1* association was validated in a second independent series of 532 FPC cases and 753 controls (p<0.0062, OR = 1.88, 95%CI 1.17–3.03). We showed segregation of the c.4249A>G *SMG1* variant in 3 affected relatives in a FPC kindred, and we found c.103G>A to be a recurrent *SMG1* variant associating with PC in both the discovery and validation series. These results suggest that *SMG1* is a novel PC susceptibility gene, and we identified specific *SMG1* gene variants associated with PC risk.

## Author summary

Pancreatic cancer (PC) remains one of the most lethal malignancies, with a 5-year survival rate of only 9%. Approximately 10% of PC occurs in families or in patients with hereditary mutations that are known to cause PC. The genetic causes of familial PC remain largely unknown. Using a new statistical method, we tested 398 patients with PC and 987 individuals without cancer (discovery series) to identify hereditary genetic variabilities that associate with PC. As a proof of principle for our methodology, we identified mutations in the *BRCA2* gene, which is known to cause PC. We also identified *SMG1* as a novel gene that associates with PC risk. To support this finding, we confirmed our observations in a separate group of 532 patients with PC and 753 individuals without cancer (validation series). In addition, we provide additional genetic evidence to support our findings by showing that a *SMG1* genetic change is present in three relatives with PC in a family. We also identified a recurrent *SMG1* variant that associated with PC in both the discovery and validation series. Our observations suggest that *SMG1* is a novel PC susceptibility gene.

## Introduction

Pancreatic adenocarcinoma (PC) remains one of the most lethal malignancies, with a 5-year survival rate of only 9%[1,2]. Since 10% of PC cases are familial (FPC) or can be accounted for by genes implicated in hereditary cancer syndromes[3,4], gene-based surveillance strategies may enable early cancer detection in at-risk individuals. However, known predisposition genes account for only a minority of FPC[4] and the hereditary basis underlying the remaining fraction of FPC remains unknown[5].

Several studies have attempted to identify the hereditary basis for the fraction of FPC unexplained by known predisposition genes[5–7]. We previously reported a candidate gene list using a filter-based approach focusing on protein truncating variants (PTVs) to prioritize candidate DNA repair genes[6]. Roberts *et al*. used a similar filter-based approach to prioritize candidate genes in a genome-wide study. Neither of these investigations identified novel genes that underlie a significant fraction of FPC[7]. In a more recent exome-wide case-control association study evaluating frequency of PTVs in 437 PC cases and 1922 controls, only *BRCA2*, the most prevalent PC predisposition gene, approached exome-wide significance (p<2.69x10^-7^) for enrichment of PTVs in PC cases[5]. These investigations highlight the heterogeneity of FPC and the challenges in delineating the genetic basis for the remaining fraction of FPC.

Region-based gene association tests may better identify genes containing rare risk variants by evaluating the combined effect of both PTVs and missense variants[5,8]. The Mixed-Effects Score Test (MiST) is a novel region-based gene association method that combines burden and variance tests to identify causal genes and can incorporate variant annotation information[9].

In this study, we searched for candidate PC susceptibility genes by examining association with both causal PTVs and missense variants. To increase statistical power, we focused on DNA damage response and repair genes as a majority of the known PC predisposition genes have a role in DNA repair. We used a rigorous approach combining MiST with a novel subsequent analysis, the leave-one-out (LOO) method, to identify potentially causal gene variants within a gene region that associates with disease[10,11]. We identified *SMG1* as a novel candidate PC susceptibility gene, which we validated in an independent case-control series.

## Materials and methods

### Ethics statement

Research ethics approval for the study was provided by the McGill University Institutional Review Board (Approval #A02-M118-11A), the University Health Network (REB 03-0049-CE, REB 12-0355-CE) and Mount Sinai Hospital (REB 03-0001-A). Written consent was provided by patients under these protocols.

### The discovery case-control series

The PC cases were collected from two individual case series, consisting of patients enrolled in either the Quebec Pancreas Cancer Study (QPCS) or the Ontario Pancreas Cancer Study (OPCS) [12,13].

The high-risk case series (Series A) consisted of 101 FPC cases (FPC; ≥2 related-individuals with PC) and 8 young onset (<50 age at diagnosis) cases, which have been previously analyzed using a filter-based approach by Smith *et al*[6]. The Montreal-Toronto case series (Series B) consisted of 289 unselected, prospectively enrolled, PC cases. Germline mutation data in *BRCA1*, *BRCA2*, *PALB2*, and *ATM* have been previously reported for Series B[14]. The controls consisted of 987 in-house samples collected from individuals without a personal history of cancer[15]. DNA from peripheral lymphocytes or whole white blood cells was isolated for sequencing as previously described[6,14].

### Candidate gene list

We evaluated the 710 cancer-related genes sequenced in Series B for a role in DNA damage response and repair based on the criteria described in the S1 Table and S1 Text. Only putative DNA damage response and repair genes (n = 445) were assessed for an association with PC risk[16,17].

### Power calculation

We calculated the power required for a simple normal Z test to identify a difference in proportions between two independent groups. As the aim was to identify a novel gene with a rare variant prevalence similar to that of *BRCA2*, the PC predisposition gene with the highest pathogenic variant prevalence[18], these calculations were based on previous estimates of *BRCA2* pathogenic variant rates in PC cases and in the general population[14,18,19]. Therefore, we estimated a pathogenic variant prevalence rate of at least 3% in PC cases, and of 0.1% in unaffected individuals. In addition, we used a case-control ratio of 1:2, given the rarity of PC cases and the likelihood of sample availability for sequencing. We calculated that a sample size of 426 cases and 852 controls would be required to detect a causal gene with 80% power following Bonferroni multiple testing correction for 445 genes (p<0.000112).

### Discovery series variant calling

**Fig 1:**
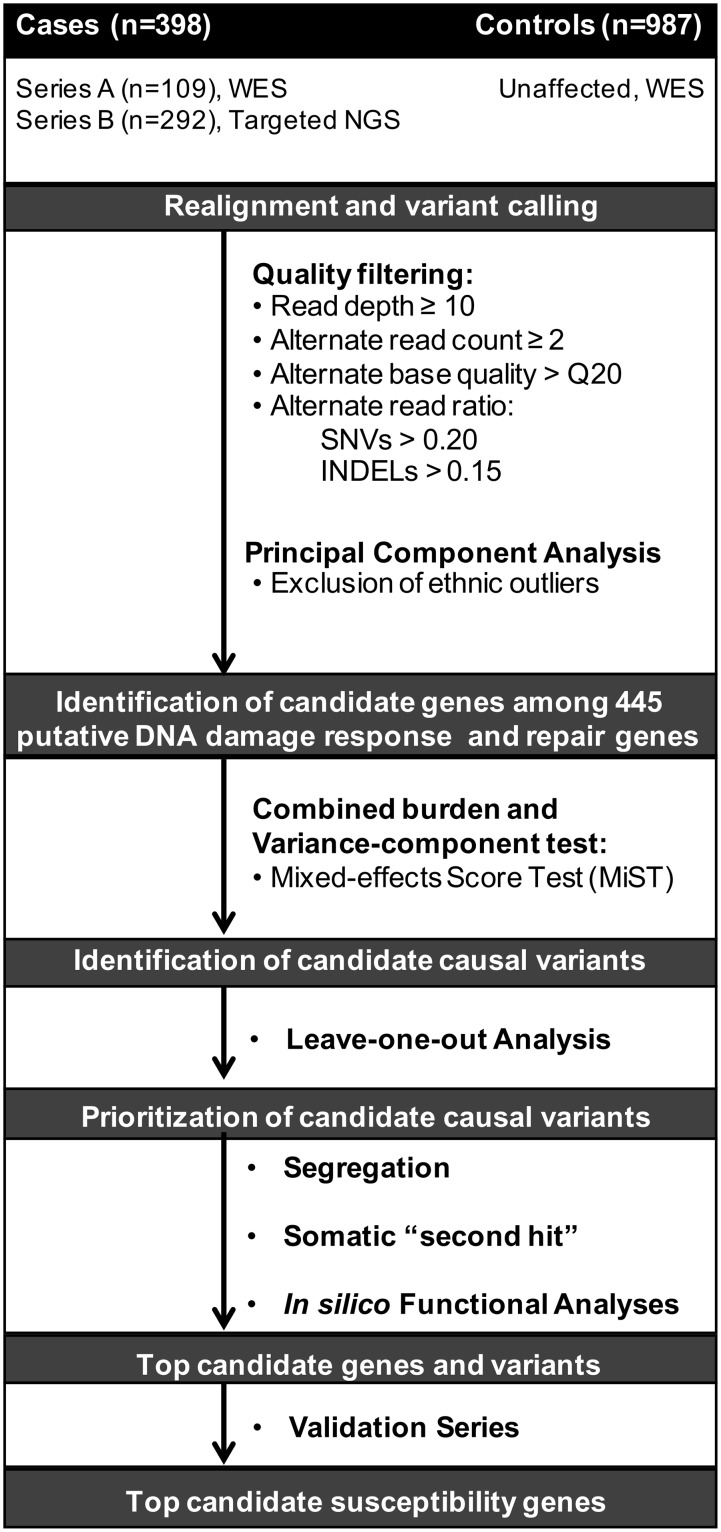
Schematic of gene association study design. Series A, cases at high risk for hereditary PC. Series B, unselected prospectively collected PC cases. WES, whole-exome sequencing. NGS, next-generation sequencing. SNVs, single nucleotide variants. INDELs, insertions/deletions.

Variants were called for all three series using a uniform pipeline and quality control filters as described in the Supplemental Materials (S1 Text and Fig 1). A principal component analysis (PCA) was performed to identify and remove individuals with mixed genetic ancestry that were more than 10 standard deviations from distinct genetic populations for the case series (S1 Text). Four individuals were identified and excluded from further analyses.

### Mixed Effects Score Test (MiST)

MiST is a gene-based test of association between a phenotype and all selected genetic variants in a region[9]. It can incorporate additional information about variants, such as the functional predictions, to give more weight to likely deleterious variants. MiST was performed using the MiST package in R (version 3.2.4) (S2 Text). Both the Combined Annotation Depletion Dependent (CADD) score for predicting variant effect on protein function[20] and the type of mutation (frameshift, non-frameshift, missense, nonsense, and splicing) were considered in our MiST analysis. Only exon and splicing variants in the 445 DNA damage response and repair genes with a depth ≥10 in at least one sample and a minor allele frequency (MAF) ≤1% in the controls were included in the analysis. Candidate genes with less than 10 variants across the case-control series were removed, as this is a threshold requirement for MiST. The MiST analysis was complemented by a leave-one-out sensitivity analysis to identify which variant was likely contributing most to any identified association (S2 Text). This analytic strategy—combining MiST with LOO analysis—has not been previously described in cancer predisposition studies. To determine the optimal MAF threshold for identifying rare variants associated with cancer predisposition, we also performed our analysis using only variants with a MAF ≤0.5%. Only 217 (3%) unique variants were removed when a MAF of ≤0.5% was applied, demonstrating a minor difference of identifying variants using these two thresholds and we selected a MAF of ≤1% for the analysis to increase the likelihood of identifying a significant association.

### Leave-one-out sensitivity analyses

The LOO method, consisting of two complementary tests, was used to identify specific variants driving the associations seen with MiST. The LOO analysis was adopted from previously described methodology [10,11].

The first test was the LOO-window (LOO-W) analysis, where each gene was split into smaller windows of 30 variants with at least a 10 variant overlap between adjacent windows. Next, each window was dropped, one at a time, and the p-value for MiST was recalculated. An increase in p-value suggested that the dropped window contained at least one risk variant. In the subsequent LOO-variant (LOO-V) analysis, we sequentially dropped each variant within the gene windows that were identified to encompass a risk variant (i.e. increase in p-value) in the LOO-W step. The p-value for MiST was recalculated after each variant was dropped. An increase in p-value ≥35% suggested that the dropped variant was driving the association identified by MiST, and it was considered a candidate pathogenic variant. The ≥35% threshold for p-value increase in the LOO-V analysis was determined using a receiver operator characteristic (ROC) curve for *BRCA2* as described in the Supplemental Materials (S1 Text).

### Characterization of candidate pathogenic variants

Segregation of candidate variants within families was assessed either through available sequencing data for related individuals or through Sanger-based genotyping of DNA isolated from peripheral lymphocytes. All missense candidate variants were assessed for loss or creation of splice sites using two *in silico* splicing prediction algorithms as described in the Supplemental Materials (S1 Text) [21,22]. Variants identified in the LOO-V analysis with a CADD score between 0–1.0 were disregarded since these variants are unlikely to alter gene function.

### Validation case-control series

The validation series consisted of 532 FPC cases, which were sequenced and processed as part of the Familial Pancreatic Cancer Sequencing Projects described by Roberts et al[7] and 753 controls from the Alzheimer’s Disease Neuroimaging Initiative (ADNI) database. Additional quality control filters were applied to decrease the false positive rate as described in the Supplemental Materials (S1 Text). A one-tailed Fisher’s exact test was used to assess for a difference in mutation frequencies between cases and controls.

The controls of the validation series used in the preparation of this article were obtained from the Alzheimer’s Disease Neuroimaging Initiative (ADNI) database (adni.loni.usc.edu). The ADNI was launched in 2003 as a public-private partnership, led by Principal Investigator Michael W. Weiner, MD. The primary goal of ADNI has been to test whether serial magnetic resonance imaging (MRI), positron emission tomography (PET), other biological markers, and clinical and neuropsychological assessment can be combined to measure the progression of mild cognitive impairment (MCI) and early Alzheimer’s disease (AD). For up-to-date information, see www.adni-info.org.

## Results

### Variants identified across 710 cancer-related genes

Across the 1385 cases and controls in the discovery series, a total of 21002 exon and splicing variants were identified in 677 genes of the 710 cancer-related genes. Of these, 8390, 11283, 477, 290, 217, and 151 variants were synonymous, missense, non-frameshift insertion/deletion (INDEL), frameshift INDEL, stop gain/stop loss, and splicing, respectively. The remaining 194 variants were identified in *PRKDC*, *UHRF1*, and *VEGFA* which were annotated in the ANNOVAR database to have an unknown effect on the protein sequence[23].

### Genetic outliers

Of the variants identified in the case series, 1703 variants had a MAF >5% and only 743 variants passed all quality-control criteria for the subsequent PCA of genetic data (S1 Text). The PCA plot for cases showed a separation of three distinct ethnic populations, representing Asian, Central/South American, and European ancestries (S1 Fig). The 31 individuals with Asian ancestry and Central/South American ancestries were not removed from further analyses since the control series was also multi-ethnic as it was collected from a comparable Canadian geographical region. However, 4 individuals that were of mixed genetic ancestry were removed. Of these, 3 were of self-reported Asian ancestry and more than 10 standard deviations (SD) away from the Asian population, while the fourth was of multiracial ancestry and more than 10 SD from any of the other populations. PCA of genetic data could not be performed for the control series as the individual genotype-level data were unavailable.

### Rare nonsynonymous variants in putative DNA repair genes

Only 7059 variants identified in 418 of the 445 putative DNA damage response and repair genes of interest remained after filtering for rare exon and splicing variants (MAF ≤1%), and excluding synonymous mutations (S1 Dataset). Of these, 6532, 149, 158, 131, and 89 variants were missense, non-frameshift INDEL, frameshift INDEL, stop gain/stop loss, and splicing respectively. The number of variants in each gene ranged from 1–99. One hundred and eighty-three of the 418 genes had <10 variants across all cases and controls and were removed from the MiST analysis, leaving 235 genes to be evaluated by MiST.

### MiST and leave-one-out analyses

Of the 235 genes tested for an association with PC risk, 48 had a p-value <0.05 (Table 1), including 3 known PC predisposition genes (i.e., *BRCA1*, *BRCA2*, and *STK11*). However, following false discovery rate analysis (R version 3.2.4), 7 genes (*ALKBH3*, *CHEK2*, *CRY2*, *PARG*, *RECQL*, *SMG1*, *TDG*) remained significant with q-values <0.05. Of these, 4 genes (*CHEK2*, *RECQL*, *SMG1 and TDG*) were significant at the Bonferroni threshold (p<0.00021) (Table 1). The LOO analyses were performed for the known PC predisposition genes significant at p-value <0.05, and for the candidate genes that remained significant following Bonferroni’s correction.

**Table 1:** List of genes with a p-value ≤ 0.05 in MiST.

Gene	p-value	q-value
*AATF*	0.01463	0.1527
ALKBH3*	0.00118	0.0404
*ANKLE1*	0.01271	0.1387
*ASTE1*	0.02218	0.1717
*ATR*	0.01008	0.1338
*AXIN2*	0.03549	0.2184
*BAZ1B*	0.00770	0.1264
*BRCA1*	0.02971	0.2128
*BRCA2*	0.01156	0.1387
*BUB1*	0.03025	0.2128
*CBFA2T3*	0.03356	0.2151
*CDC25B*	0.00288	0.06287
*CDH1*	0.02212	0.1717
*CEP164*	0.03918	0.2187
***CHEK2***	**0.00018**	0.01053
*CRB2*	0.04734	0.2417
*CREBBP*	0.01572	0.1528
CRY2*	0.00032	0.01287
*DDX1*	0.03344	0.2151
*ERCC3*	0.04628	0.2415
*FANCM*	0.00286	0.06287
*JMY*	0.02609	0.1957
*LIG1*	0.03406	0.2151
*MLH3*	0.02034	0.1717
*MUM1*	0.02130	0.1717
*NEK1*	0.03719	0.2187
*NEK11*	0.03889	0.2187
PARG*	0.00024	0.01147
*POLE*	0.03103	0.2128
*POLG*	0.04856	0.2428
*POLL*	0.00902	0.1273
*RAD9A*	0.02094	0.1717
*RASSF1*	0.01592	0.1528
*RBM14*	0.00843	0.1264
***RECQL***	**0.00016**	0.01053
*RECQL4*	0.01820	0.1680
*RFWD2*	0.01059	0.1338
*SETD2*	0.04173	0.2226
*SMC5*	0.04089	0.2226
***SMG1***	**3.22E-7**	7.73E-05
*STK11*	0.03889	0.2187
***TDG***	**0.00017**	0.01053
*TET1*	0.00735	0.1264
*USP1*	0.00556	0.1113
*UVRAG*	0.00270	0.06287
*WRN*	0.00234	0.06287
*XAB2*	0.00791	0.1264
*XPA*	0.01238	0.1387

The 48 DNA repair genes with a significant association (p ≤ 0.05) in MiST and their corresponding q-values are listed. Bolded genes have a p-value that is significant following multiple testing correction by Bonferroni (p ≤ 0.00021). Genes with an asterisk have a q-value <0.05, but were not significant at the Bonferroni significance threshold.

**Fig 2:**
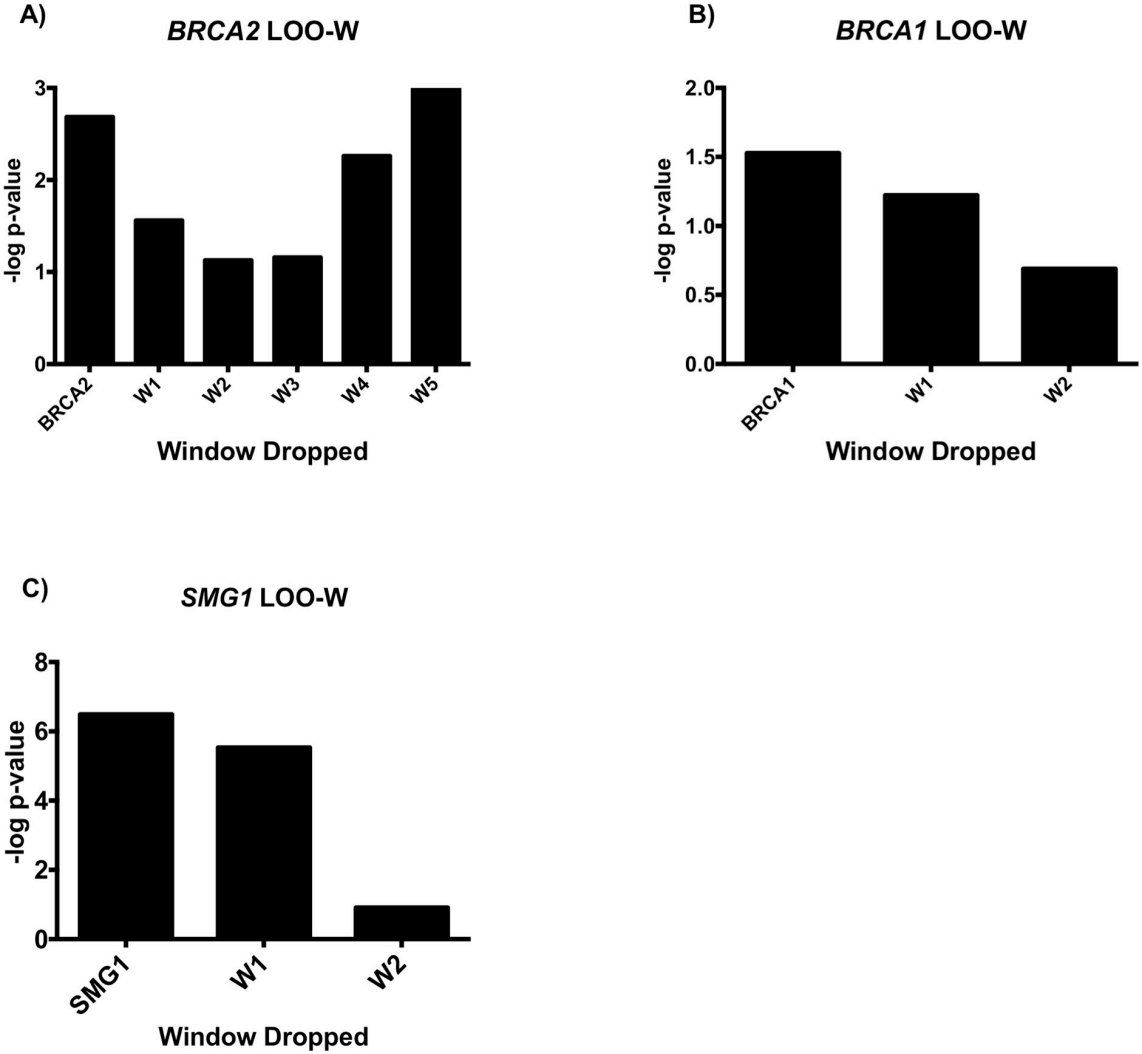
The–log p-value graphs for the LOO-W analysis for *BRCA2*, *BRCA1* and *SMG1*. A decrease in the–log p-value is an increase in p-value signifying the window dropped contains variants driving the association with PC risk. Any window with an increase in p-value was analyzed by LOO-V for potential variants of interest. A) LOO-W for *BRCA2*. B) LOO-W for *BRCA1*. C) LOO-W for *SMG1*.

**Fig 3:**
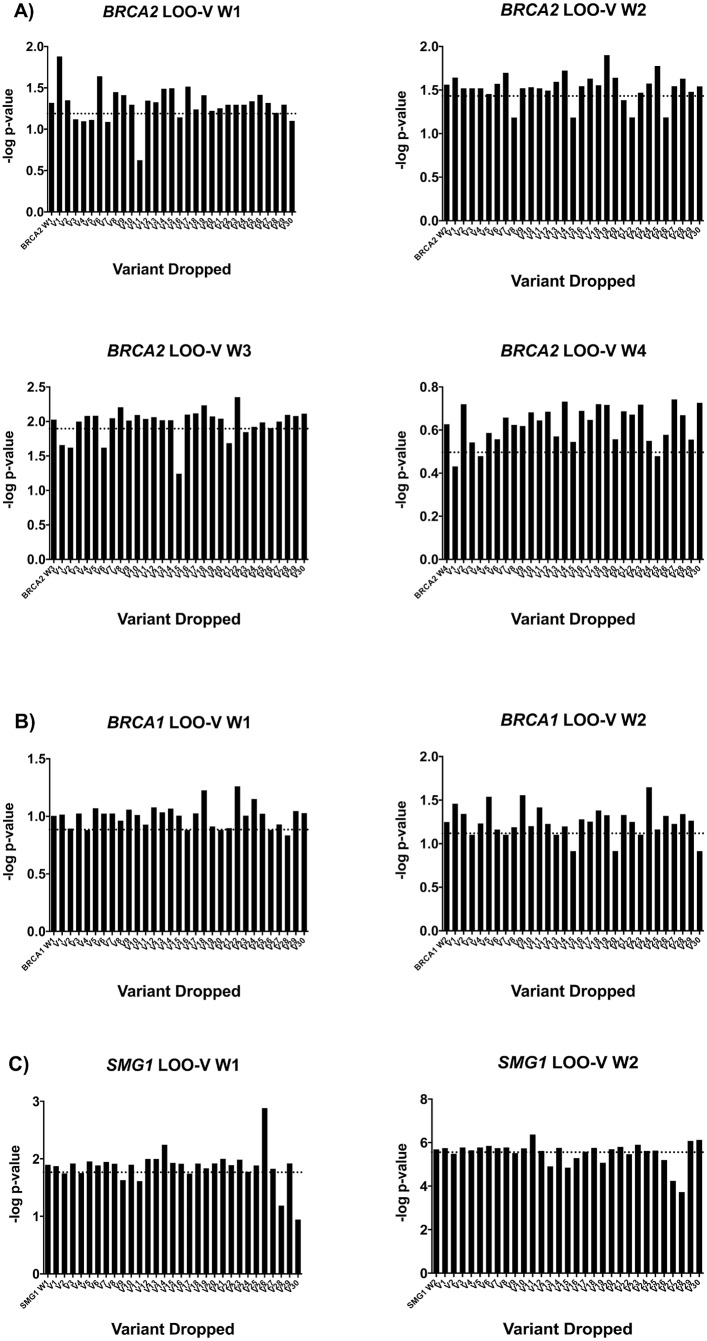
The–log p-value graphs for LOO-V analysis for each significant window for *BRCA2*, *BRCA1*, *SMG1*. A decrease in the–log p-value corresponds to an increase in p-value, signifying the dropped variant is potentially driving the association with PC risk. The dotted line represents the–log p-value for an increase in p-value of 35% compared with the p-value of the window. This is the threshold for identifying a candidate variant. A) LOO-V for window 1 to window 4 of *BRCA2*. B) LOO-V for window 1 and window 2 of *BRCA1*. C) LOO-V for window 1 and window 2 of *SMG1*.

We first performed a ROC curve analysis using variants identified in *BRCA2* to determine the threshold for p-value increase that would provide the highest sensitivity and specificity for the LOO analyses (S2 Fig). Since the p.K3326X stopgain variant has not been shown to result in loss of protein function, it was excluded from the analysis. Across all samples, 96 unique *BRCA2* variants were identified. The gene was split into 5 windows with 30 variants in each window and a minimum overlap of 10 variants. The first 4 windows (spanning variants 1–30, 21–50, 41–70, 61–90) led to an increase in MiST p-value when dropped (Fig 2A). Thus, the LOO-V analysis was performed for these 4 windows (Fig 3). Using the ROC curve for the LOO-V analysis, we determined that an increase in p-value for MiST of at least 35% when a given variant was dropped results in a sensitivity of 100% (95% CI 66–100%) and a specificity of 88% (95% CI 78–94%) for identifying pathogenic variants. At this threshold, 19 unique variants in 25 cases and in 1 control were identified as driving the association with PC risk (Table 2). Therefore, the 35% p-value increase threshold was used for the LOO-V analysis of the remaining genes: *BRCA1*, *STK11*, *SMG1*, *RECQL*, *TDG* and *CHEK2*.

**Table 2:** Summary of mutations identified in the discovery series by the leave-one-out analysis for 2 known susceptibility genes and a candidate PC susceptibility gene.

Gene	Chromosomal Position	Mutation	Type	CADD	Case MAF	Control MAF	EVS	ExAC	1000s	HSF/ MES	p-value increase (%)
*BRCA2*	13:32893369	c.223G>C:p.A75P	Missense	23.8	0.0026	0.0005	3.1E-4	1.6E-4	.	.	>55%
*BRCA2*	13:32900750	c.631G>A:p.V211I	Missense	26	0.0013	0	.	.	.	BD	>65%
*BRCA2*	13:32903604	**c.658_659delGT:p.V220fs**	Frameshift	24.3	0.0013	0	.	4.9E-5	.	.	>55%
*BRCA2*	13:32906541	**c.927delA:p.S309fs**	Frameshift	26.4	0.0013	0	.	.	.	.	>65%
*BRCA2*	13:32906766	c.1151C>T:p.S384F	Missense	23.4	0.0039	0	1.1E-3	6.8E-4	.	.	>105%
*BRCA2*	13:32907395	c.1780A>T:p.I594L	Missense	20.5	0.0013	0	.	1.6E-5	.	.	>45%
*BRCA2*	13:32911601	**c.3109C>T:p.Q1037X**	Stopgain	37	0.0013	0	.	.	.	.	>105%
*BRCA2*	13:32912060	c.3568C>T:p.R1190W	Missense	25.3	0.0013	0	.	1.1E-4	.	.	>65%
*BRCA2*	13:32912663	**c.4171G>T:p.E1391X**	Stopgain	41	0.0013	0	.	.	.	.	>105%
*BRCA2*	13:32913077	c.4585G>A:p.G1529R	Missense	29.2	0.0026	0	4.6E-4	4.2E-4	.	CA	>105%
*BRCA2*	13:32913182	**c.4691dupC:p.A1564fs**	Frameshift	25.6	0.0013	0	8E-5	.	.	.	>105%
*BRCA2*	13:32913554	**c.5064dupA:p.E1688fs**	Frameshift	34	0.0013	0	.	.	.	.	>105%
*BRCA2*	13:32914437	**c.5946delT:p.S1982fs**	Frameshift	35	0.0026	0	.	2.6E-4	.	.	>105%
*BRCA2*	13:32918706	c.6853A>G:p.I2285V	Missense	25.5	0.0026	0	2.3E-4	2.7E-4	.	CA	>105%
*BRCA2*	13:32920979	c.6953G>A:p.R2318Q	Missense	35	0.0013	0	.	1.6E-5	.	.	>45%
*BRCA2*	13:32928996	**c.7008-2A>T**	Splicing	23	0.0013	0	.	.	.	.	>35%
*BRCA2*	13:32936782	c.7928C>G:p.A2643G	Missense	32	0.0013	0	7.7E-5	2.5E-5	.	.	>35%
*BRCA2*	13:32937618	c.8279G>T:p.G2760V	Missense	31	0.0013	0	.	.	.	CD	>35%
*BRCA2*	13:32950851	**c.8677C>T:p.Q2893X**	Stopgain	51	0.0013	0	.	.	.	.	>35%
*BRCA1*	17:41276044	**c.66_67del:p.L22fs**	Frameshift	32	0.0013	0	.	2.2E-4	.	.	>105%
*BRCA1*	17:41245422	**c.2125_2126insA:p.F709fs**	Frameshift	22.3	0.0013	0	.	.	.	.	>105%
*SMG1*	16:18908268	c.103G>A:p.A35T	Missense	21.8	0.0088	0.0005	0.0027	0.019	.	.	>105%
*SMG1*	16:18908112	c.256+2delGA	Splicing	24.1	0.0039	0	.	.	.	.	>105%
*SMG1*	16:18908113	c.256+2delTC	Splicing	24.1	0.0013	0	.	.	.	.	>105%
*SMG1*	16:18887501	c.1835T>A:p.I612K	Missense	17.25	0.0013	0	.	0.043	.	.	>65%
*SMG1*	16:18881315	c.2494A>G:p.N832D	Missense	12.03	0.0026	0	.	0.0021	.	.	>105%
*SMG1*	16:18872021	c.3773A>G:p.N1258S	Missense	4.325	0.0013	0	.	4.2E-5	.	CA	>105%
*SMG1*	16:18870914	c.3917C>T:p.P1306L	Missense	8.947	0.0052	0.0005	0.0018	0.0017	.	.	>105%
*SMG1*	16:18866212	c.4249A>G:p.I1417V	Missense	6.2	0.0026	0	.	1.7E-5	.	CD/CA	>105%
*SMG1*	16:18863489	c.4952C>G:p.S1651C	Missense	19.77	0.0013	0	8.4E-5	2.0E-4	.	CD	>45%
*SMG1*	16:18848732	c.7447G>A:p.V2483I	Missense	18.38	0.0013	0	.	.	.	.	>55%
*SMG1*	16:18844389	c.8665G>A:p.G2889S	Missense	11.67	0.0013	0	.	0.0013	.	CA	>85%
*SMG1*	16:18841066	c.9145C>T:p.L3049F	Missense	12.59	0.0013	0	.	.	.	.	>85%
*SMG1*	16:18823386	c.10685T>C:p.L3562P	Missense	18.78	0.0013	0	.	.	.	.	>35%
*SMG1*	16:18820956	c.10921A>G:p.I3641V	Missense	18.52	0.0013	0	.	.	.	.	>35%

The variants correspond to the following transcripts: *BRCA2* NM_000059, *BRCA1* NM_007294, and *SMG1* NM_015092. MAF for our case series, control series, and 3 public databases as well as the CADD score are shown. The p-value increase observed for the leave-one-out variant test and the prediction on splicing for missense variants are indicated. CADD, combined annotation depletion dependent. MAF, minor allele frequency. EVS, Exome Variant Server. ExAC, Exome Aggregation Consortium. 1000s, 1000 genomes project. HSF, Human Splicing Finder. MES, MaxEntScan. BD, broken splice donor site. CA, creation of splice acceptor site. CD, creation of splice donor site.

The LOO analyses revealed that the *STK11*, *RECQL*, *TDG* and *CHEK2* associations were driven by more variants identified in the control series rather than the case series (S2 Table). Therefore, these genes were not considered further. It is of course possible that these variants have a protective effect against PC risk.

There were 44 unique variants identified in *BRCA1*, which was split into 2 windows (spanning variants 1–30, 15–44) for the LOO-W analysis (Fig 2B). Both windows had an increase in p-value. Thus, LOO-V was performed for both windows. Seven variants were identified in 8 cases, including two known pathogenic frameshift variants (Fig 3, Table 2).

In *SMG1*, 45 unique variants were identified in 41 cases (10.3%) and 45 controls (4.6%). The gene was split into two windows for the LOO-W analysis (spanning variants 1–30 and 16–45) and an increase in p-value was observed for both windows (Fig 2C). Subsequent LOO-V analyses for both windows identified 14 unique variants across 27 cases and 2 controls driving the association with PC risk (Fig 3). Of these variants, 12 were missense and 2 were splicing variants (Table 2). The clinical characteristics for the 27 individuals carrying one of these 14 variants are detailed in Table 3.

**Table 3:** Clinical characteristics of carriers of the 14 *SMG1* variants identified in LOO-analysis.

ID	Variant	Sex	Ethnic Ancestry	Age of PC diagnosis (in years)	Stage at diagnosis	Other cancer diagnoses with age of diagnosis (in years)
A-26	**c.1835T>A (p.I612K)**	M	European	70	IIA	.
A-78	**c.4249A>G (p.I1417V)**	M	European	53	III	.
A-79	**c.4249A>G (p.I1417V)**	F	European	75	IV	Basal Cell Carcinoma, 65, Squamous Cell Carcinoma, 66
A-100	**c.3917C>T (p.P1306L)**	M	European	77	III	Skin (unknown type), 70
B-8	**c.256+2delTC**	F	European	78	IV	Breast 48, 63, Bladder 61
B-11	**c.3917C>T (p.P1306L)**	M	European	70	IV	.
B-17	**c.8665G>A (p.G2889S)**	M	Asian	67	IV	.
B-24	**c.256+2delGA**	M	European	75	IV	.
B-35	**c.256+2delTC**	F	European	58	III	.
B-75	**c.10685T>C (p.L3562P)**	M	European	70	III	.
B-95	**c.3917C>T (p.P1306L)**	F	European	51	IV	.
B-99	**c.103G>A (p.A35T)**	M	European	61	IV	.
B-105	**c.4952C>G (p.S1651C)**	F	European	83	IV	.
B-149	**c.9145C>T (p.L3049F)**	M	Asian	65	IV	.
B-165	**c.103G>A (p.A35T)**	M	Asian	58	IIB	.
B-166	**c.103G>A (p.A35T)**	M	Asian	70	I-III	.
B-168	**c.10921A>G (p.I3641V)**	M	European	49	IV	.
B-191	**c.103G>A (p.A35T)**	F	Asian	76	III	.
B-207	**c.103G>A (p.A35T)**	M	Asian	79	IIA	.
B-221	**c.3773A>G (p.N1258S)**	F	Asian	76	II	.
B-242	**c.103G>A (p.A35T)**	F	Asian	64	III	.
B-247	**c.103G>A (p.A35T)**	F	European	76	IIB	Melanoma, 75
B-253	**c.2494A>G (p.N832D)**	F	European	60	III	.
B-259	**c.7447G>A (p.V2483I)**	F	European	92	IV	.
B-263	**c.2494A>G (p.N832D)**	M	Central/South American	72	IV	.
B-276	**c.3917C>T (p.P1306L)**	M	European	69	IV	.
B-281	**c.256+2delTC**	F	European	63	II	.

The ethnic ancestry column denotes the ancestry group the individual clustered with in the PCA. M, male; F, female.

### Validation of *SMG1* in FPC case-control series

To provide further evidence for *SMG1* as a novel PC predisposition gene, we validated our findings in an independent case-control series consisting of 532 FPC cases (defined as ≥2 first-degree relatives with PC) and 753 non-cancer controls. We observed non-synonymous *SMG1* variants in 41 (7.71%) FPC cases and in 32 (4.24%) controls (p<0.0062, OR = 1.88, 95% CI 1.17–3.03) (S3 Table).

Interestingly, the nonsynonymous variant c.103G>A (p.A35T) was observed at a higher frequency in cases *versus* controls in both the discovery (p = 0.0009) and validation (p = 0.012) series, suggesting that it may be a recurrent *SMG1* variant associated with PC risk. Since this variant is enriched in some ethnic populations, particularly the East Asian and Latino populations with a reported MAF of 13.3% and 9.4% in the Genome Aggregation Database (gnomAD)[24], we assessed the difference in variant frequency for only the cases with European Ancestry. The allele frequency for non-Finnish Europeans observed in the non-cancer samples in gnomAD is 0.38% (278/73592) compared with the observed allele frequency of 0.95% (17/1798) for all European PC cases from both the discovery and validation series (p = 0.0001).

### Evaluation of variants in *BRCA1*, *BRCA2*, and *SMG1*

We first evaluated the list of variants in the 2 known predisposition genes, *BRCA1* and *BRCA2*. Excluding the known pathogenic variants, there were 5 and 8 unique missense variants in *BRCA1* and *BRCA2*, respectively. However, the 5 missense variants in *BRCA1* were discarded as they had a CADD score between 0–1.0. The 8 missense variants in *BRCA2* were observed in 13 cases and 1 control (Table 2). Unfortunately, we were unable to further validate these variants as tumour tissue was unavailable for these cases to determine whether there was a somatic second hit. There was no opportunity for segregation studies as no samples were available from their blood relatives.

**Fig 4:**
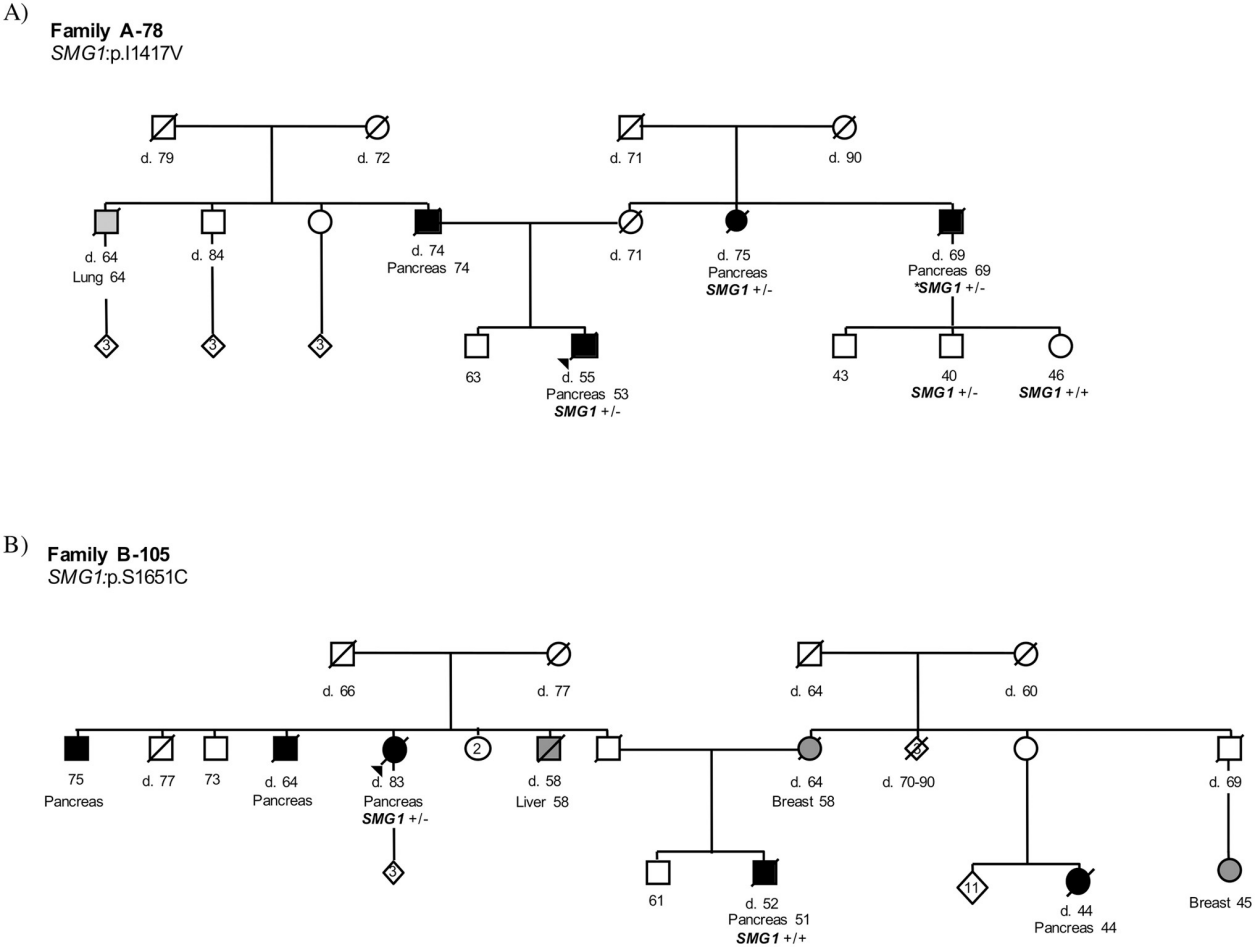
Pedigrees for two *SMG1* variant carriers with segregation opportunities. A) In family A-78, both the proband and the maternal aunt were included in our case series and were identified to be carriers of the c.4249A>G (p.I1417V) *SMG1* variant. *SMGI c.4249A>G carrier status for the maternal uncle with PC was inferred by genotyping his son. B) In family B-105, the maternal aunt was included in our case series and was identified to be a carrier of the c.4952C>G (p.S1651C) *SMG1* variant. The proband was found to not be a carrier of this *SMG1* mutation.

Although there were no tumour samples available to test for somatic inactivating second hit mutations, lymphocyte DNA was available to evaluate for segregation of the *SMG1* variants with PC in two families with European ancestry (Table 2). For the first family (A-78), the c.4249A>G (p.I1417V) variant was identified in two related individuals in our case series, the proband and the maternal aunt (Fig 4A). We were then able to confirm the mutation in one of two maternal first cousins whose father had PC and, by inference, the latter affected patient also carried the c.4249A>G variant. Thus, the c.4249A>G variant segregated in all 3 individuals with PC on the maternal side. In the second family (B-105), there was a history of PC on both the maternal (1 relative) and paternal (3 relatives) sides of the family (Fig 4B). The c.4952C>G (p.S1651C) variant was identified in the paternal aunt in our case series. However, it did not segregate in the proband with PC, possibly representing disease phenocopies in the family. Unfortunately, samples from the other paternal relatives with PC and their children were not available to determine whether the *SMG1* variant segregated with PC on the paternal side of the family.

To further evaluate the functional consequence of the *SMG1* variants that emerged following the LOO-V analysis, we performed *in-silico* splicing prediction analyses for all missense variants in *SMG1* (Table 2). Interestingly, the c.4249A>G variant identified in family A-78, which segregated with two relatives with PC, was predicted to create both a splice acceptor and splice donor site. In addition, the c.4952C>G variant observed in family B-105 was also predicted to create a splice donor site.

## Discussion

Challenges in identifying novel PC predisposition genes may be explained by the genetic heterogeneity of familial PC[7]. In a recent case-control exome-wide association study of 437 PC cases and 1922 non-cancer controls, only *BRCA2* approached significance for enrichment of rare inactivating variants in PC cases[5]. The authors concluded that, due to the genetic heterogeneity of familial PC, large cohorts with novel statistical methods will be required to identify novel predisposition genes. Another important finding is that the majority of genes associated with PC risk are DNA repair genes[25,26]. Therefore, we focused the current study on putative DNA damage response and repair genes, and applied a novel statistical approach combining MiST with the LOO method to identify novel genetic variants associated with PC risk.

Region-based genetic association tests compare variants within a gene or a gene region in cases *versus* controls to predict whether a gene is associated with increased risk[8]. MiST is a region-based association test that incorporates a hierarchical-based model to account for confounders and predictive protein functionality scores of variants[9]. Moreover, MiST has been used successfully to identify genetic associations with complex traits[8,9], while the LOO analysis has been successfully combined with region-based association tests to identify causal variants[10].

Since MiST in combination with the LOO analysis had not been previously used in cancer predisposition studies, we performed the analyses at two MAF thresholds (≤1% and ≤0.5%). At both thresholds, *BRCA1* and *BRCA2* were associated with PC risk. The p-values at the ≤1% MAF threshold for *BRCA1* and *BRCA2* were 0.0297 and 0.0016, respectively. However, following Bonferroni’s correction for multiple testing (p<0.000112), the association was lost for both genes. The pathogenic mutation frequency of *BRCA1* in PC is 0.5%-1% in populations unaffected by a founder effect[14,19]. Since our study was designed to identify genes with a pathogenic mutation frequency of 3%, we did not expect to identify an association with *BRCA1*. Similarly, we did not expect to observe an association with other known PC predisposition genes that carry a mutation frequency in PC of <3% (e.g., *PALB2*, *ATM*)[5,14,27]. However, the study was designed to detect an association of genes that carry a cumulative pathogenic variant frequency of at least 3%, including *BRCA2* which has a 3–5% reported frequency of germline mutations in PC [5,14,19]. Loss of the *BRCA2* association following correction for multiple testing may be explained by the exclusion of known germline *BRCA2* mutations in Series A that formed part of the discovery case series[6].

As a proof of principle, we used a ROC curve to determine the p-value change threshold required to identify known pathogenic mutations in *BRCA2*. At a p-value increase threshold ≥35%, we were able to identify all pathogenic mutations with a sensitivity of 100% (95% CI 66–100%) and a specificity of 88% (95% CI 78–94%). In addition to the known pathogenic mutations, novel potentially causal missense variants were identified. Unfortunately, samples were not available for segregation studies of these missense variants in affected relatives and to assess for somatic inactivation of the second *BRCA2* allele in the corresponding tumours.

Following correction for multiple testing, *SMG1* was the only gene with a significant p-value (p = 3.22x10^-7^) that was driven by variants in PC cases. The variant frequency in cases was 10.3% *versus* 3.6% in controls. Interestingly, only two PTVs, both splicing variants (c.256+2delGA and c.256+2delTC), and one non-frameshift variant (c.34_36delGCT) were identified. The other 42 unique variants identified were all missense changes. This observation is in keeping with the *SMG1* genetic alterations in the COSMIC database[28]. There are no *SMG1* PTVs in COSMIC PC cases and *SMG1* PTVs are rarely present in other cancer types (50/42739 samples; 0.12%).

SMG1 is a serine/threonine protein kinase in the same protein family as ATM[29]. SMG1 is implicated in p53 regulation following genotoxic stress and in nonsense-mediated mRNA decay (NMD)[30]. Loss of SMG1 function has also been associated with tumorigenesis[30,31]. Gubanova *et al*. observed decreased p53 activity following ionizing radiation in U2-OS cells with loss of SMG1 compared to SMG1-wildtype cells, resulting in increased cell proliferation[30]. This study also showed that SMG1-deficient cells are unable to induce degradation of CDK2, a cell cycle checkpoint protein, and downregulation of Cdc25a, a related cell cycle checkpoint protein, leading to increased CDK2 activation and cell cycle progression after exposure to genotoxic stress. *Gubanova et al*. also knocked down *SMG1* expression in HA1EB cells using shRNA and found that mice with *SMG1* knockdown developed tumours more rapidly compared to mice with unaltered *SMG1* expression. Furthermore, Roberts *et al*. showed that mice with only one functional *SMG1* allele are more likely to develop papillary lung adenocarcinoma[31]. These observations suggest that *SMG1* may have a role as a tumor suppressor gene.

**Fig 5:**
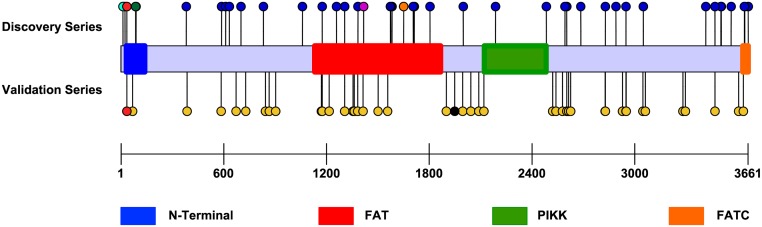
A lollipop diagram depicting the mutation landscape for *SMG1* variants identified in both the discovery and validation series. *SMG1* consists of 4 main functional domains represented by the blue (N-terminal domain—amino acid (aa) 32–140), red (FAT domain—aa 1131–1866), green (PIKK domain—aa 2121–2482), and orange (FATC domain—aa 3629–3661). Variants identified in the discovery series are depicted by the upward lollipops and variants identified in the validation series are depicted by the downward lollipops. The red lollipop located in the N-terminal domain represents the recurrent p.A35T variant identified in both the discovery and validation series. The purple and orange lollipops located in the FAT domain represent the p.I1417V variant, which segregated in kindred A-78, and the p.S1651C variant identified in kindred B-105, respectively. The dark green and black lollipops represent the amino acid position adjacent to the splicing variants identified. The light blue lollipop represents the non-frameshift variant identified.

In both the discovery and validation series, variants were identified across the entire gene (Fig 5). Similar to other serine/threonine protein kinases, *SMG1* consists of 4 major domains: the N-terminal, FAT (FRAP, ATM and TRRAP), PIKK (PIK-related kinase), and FATC (FAT carboxyl terminus) domains[32–35]. A majority of variants (8/14) identified in the LOO-V analysis were localized to the four functional domains, including the recurring variant (p.A35T) and the variant that segregated in kindred A-78 (p.I1417V) (Fig 5).

One limitation of our case-control series is a potential bias introduced by the inclusion of related individuals (101 individuals from 85 families) in Series A of the discovery series, which may inflate the frequency of rare variants. In addition, as individual genotype-level data for the controls were not available for PCA analysis to remove genetic outliers, we were unable to confirm that the proportion of ethnic populations were similar between cases and controls. A difference in proportions may inflate the frequency of rare variants that are unique to specific ethnic populations. These limitations were, however, addressed by validating the *SMG1* association in an independent case-control series. In addition to being a second unrelated case-control series, the validation series did not include related individuals or non-Caucasians. The observation of a significant association (p<0.0062) of *SMG1* variants in cases (41/532; 7.7%) *versus* controls (32/754; 4.2%) in the validation series provides further support for *SMG1* as a candidate PC susceptibility gene.

Segregation of the c.4249A>G variant with PC in kindred A-78 (Fig 4a) provides additional evidence for *SMG1* as a PC predisposition gene. This nonsynonymous variant is predicted to affect splicing, and segregated with all 3 PC cases on the maternal side of the family. There was opportunity for segregation assessment in only one other family. This kindred, B-105, harboured the c.4952C>G variant, which was identified in the proband’s paternal aunt who had PC and was included in Series A of the discovery case series. The one relative with PC that we could test for segregation was the proband, but he did not carry the variant (Fig 4B). However, the lack of segregation may be explained by the proband’s tumour being a phenocopy. Moreover, as this kindred has affected relatives on both the paternal and maternal sides of the family, the genetic predisposition may be originating from the paternal side. In support of this possibility is that the c.4952C>G variant segregates to the paternal side, which has 3 affected relatives in the same generation rather than the single affected relative on the maternal side (Fig 4B). Finally, the presence of a recurrent variant (i.e., c.103G>A) that associates with PC, in both the discovery and validation series, provides further support for the causal role of *SMG1*. While this variant may not alter protein function given the enriched MAF in the Asian and Latino populations and the presence of homozygotes in gnomAD, this variant may be in linkage disequilibrium with a pathogenic variant in Europeans, resulting in the association with PC observed in this European population. Alternatively, there may be a protective genetic variation among Asian and Latino populations that balances the penetrance of the c.103G>A in these populations.

In summary, we used a novel approach by combining MiST with the LOO analysis to identify causal genetic drivers of a familial cancer plagued by genetic heterogeneity. Specifically, we investigated for novel susceptibility genes with a significant contribution to familial PC by using a mutation frequency of at least 3% based on the germline mutation prevalence of *BRCA2*, the most common known PC predisposition gene. We validated this methodology by identifying pathogenic *BRCA2* mutations, and identified *SMG1* as a novel PC predisposition gene.

## Supporting information

S1 Dataset Summary variant table. List of rare variants (MAF<1%) identified for gene association test with observed allele frequencies in cases and controls for discovery series.
(XLSX)

S1 Text Supplemental materials. Additional methods and materials.
(DOCX)

S2 Text Code for MiST and LOO analyses. Code for R used for the primary MiST analyses and sample code for LOO analyses used for *SMG1*.
(PY) Click here for additional data file.

S1 Fig PCA plot for discovery case series. Exon and splicing variants with a MAF >5% in 710 cancer-related genes. Blue dots represent individuals that clustered in the European ancestry group, green dots represent individuals that clustered in the South/Central American ancestry group, and red dots represent individuals that clustered in the Asian ancestry group. Individuals of mixed ancestry indicated by the purple dots were removed from further analyses.
(TIFF)

S2 Fig ROC curve for the *BRCA2* leave-one-out analysis. Solid line represents the sensitivity and false positive rate for different p-value increase thresholds (5%-105%) for the LOO-V analysis for *BRCA2*. The dotted line represents the identity line for a 50/50 test.
(TIFF)

S1 Table List of 710 cancer-related genes sequenced in Series B of the discovery series. (DOCX)

S2 Table Summary of variants identified in the discovery series by the leave-one-out analysis for 4 genes with a significant association in MiST. The majority of variants identified in these 4 candidate genes were identified in the control series and were thus excluded from further analyses. MAF for our case series, control series, and 3 public databases as well as the CADD score are shown. The p-value increase observed for the leave-one-out variant test is indicated. CADD, combined annotation depletion dependent. MAF, minor allele frequency. EVS, Exome. Variant Server. ExAC, Exome Aggregation Consortium. 1000s, 1000 genomes project.
(DOCX)

S3 Table *SMG1* variants identified in the validation series. The CADD score and minor allele frequency (MAF) for each variant in the case series, in the controls and in the 3 public databases are shown. CADD, combined annotation depletion dependent. EVS, Exome. Variant Server. ExAC, Exome Aggregation Consortium. 1000s, 1000 genomes project. LOO-V, leave-one-out variant analysis.
(DOCX)
