# Supplementary material for: A region-based gene association study combined with a leave-one-out sensitivity analysis identifies SMG1 as a pancreatic cancer susceptibility gene
Source: PLoS Genet. 2019 Aug 30;15(8):e1008344. doi: 10.1371/journal.pgen.1008344 (PMC6742418; doi:10.1371/journal.pgen.1008344)
Supplement: S1 Text — Additional methods and materials. (DOCX) [file pgen.1008344.s002.docx]

**Supplemental Methods**

***Candidate gene list:***

Series B was sequenced using a 710 cancer-related targeted gene panel and we focused our analysis on genes defined as being implicated in DNA damage response and repair. The Database for Annotation, Visualization and Integrated Discovery (DAVID) pathways, and gene ontology (GO) level 4 and level 5 were used to identify the cellular pathway implications of each of these 710 genes[1,2]. We identified 378 genes implicated in DNA repair (HDR, NHEJ, MMR, NER, BER, TLS), DNA damage response and checkpoints, end processing of break sites, and modification of DNA repair proteins. A subsequent literature review for the remaining 332 genes suggested 67 of these 332 genes may also have a role in DNA repair[2,3]. Therefore, we identified 445 genes with a putative role in DNA damage response and repair to interrogate for an association with PC risk.

**Variant calling**

Raw sequencing data (FASTQ) files were obtained for both case series and processed through the same pipeline as the control series, which was provided as processed and filtered Variant Call Format (VCF) files. Burrows Wheeler Alignment was used to align reads to the reference genome (Hg19)[4]. Picard was used for converting files from the SAM format to BAM, sorting and indexing of the BAM files, and to mark duplicate reads[5]. The Genome Analysis Tool Kit (GATK) package was used to remove duplicate reads, and for the realignment of insertions and deletions (INDELs)[5]. The samtools module, mpileup, was used for variant calling to call single nucleotide variations (SNVs) and small INDELs[5]. Variants were only considered with a depth≥3, base quality≥20, and the alternate allele present in at least ≥15% of reads.

***Variant annotation:***

The following databases were used through ANNOVAR to annotate the variants. The RefSeq database was used to annotate gene names, location of variation (e.g., exonic), and type of mutation (e.g., nonsynonymous)[6]. The Exome Aggregate Consortium (ExAC), Exome Variant Server (EVS), and 1000 genomes project (1000s) were used to determine the MAF of variants in the public database[6]. The Combined Annotation Dependent Depletion (CADD) database was used to determine predictive pathogenicity scores for each variant, including the scores for Polyphen-2, Sorting Intolerant from Tolerant (SIFT), and Genomic Evolutionary Rate Profiling (GERP)[7].

***Principal Component Analysis (PCA):***

A principal component analysis (PCA) was performed to determine whether any individual sample in the case series (A and B) was genetically diverse from the rest[8]. Only exonic variants identified in the 710 genes sequenced in the Series B with a minor allele frequency (MAF) > 5% were included in the analysis. In addition, variants that were present in only one case series (A or B) were excluded due to difference in coverage between the two sequencing platforms. A total of 743 variants passed these criteria. A PCA plot of principal component (PC) 1 and PC 2 was used to determine which cases were >10 standard deviations from their respective ethnic populations (Supplemental Figure 1). These cases were excluded from further analyses.

***Receiver Operator Curve (ROC):***

To determine a significant threshold for a p-value increase in the LOO-V test, we performed a ROC curve analysis for *BRCA2* as a proof of principle (Supplemental Figure 2). Among the 394 cases, 9 pathogenic mutations in *BRCA2* were identified. Sensitivities and specificities were calculated for different thresholds (5%-105% increase in p-value at intervals of 10%), under the assumption that all variants, except for the known pathogenic mutations, were not associated with PC risk.

***In Silico splicing analyses:***

All missense variants were assessed for loss/creation of splice sites using two *in silico* splicing prediction algorithms: Human Splicing Finder (HSF) and MaxEntScan[9,10]. For HSF, a score >65 was considered to identify a functional splice site, and a variant that results in loss/creation of a splice site with a score difference of >10% between the wild-type and variant genotype was predicted to affect splicing. Similarly, MaxEntScan considers a variant with a score >3 with a score difference > 20% between wild-type and variant genotype to affect splicing.

***Validation Case-Control Series:***

The validation control-series consisted of the FPC case series previously reported by Roberts *et al.* and the 753 controls. The OPCS and QPCS cases that were included in the Roberts *et al.* FPC series and also included in discovery series A were removed from the validation analysis[11]. The following filters were used to decrease the false positive rate. First, samples with a non-reference discordance rate >5% or mismatched sex were removed. Based on clustering of cases and controls along with the HapMAP phase 3 populations, non-European samples were excluded (27 cases and 51 controls). Using the Genome-wide complex trait analysis (GCTA) program, an individual from sample pairs with an estimated relatedness score greater than 0.05 was excluded (42 cases and 4 controls). Following quality control for samples, variants were filtered to have a genotype quality score ≥20 and depth ≥10. In addition, monomorphic variants and variants with missingness >10% were excluded.

1. The Gene Ontology Consortium. Expansion of the Gene Ontology knowledgebase and resources. Nucleic Acids Research. 2017 Jan 4;45(D1):D331–8.

2. Ruark E, Snape K, Humburg P, Loveday C, Bajrami I, Brough R, et al. Mosaic PPM1D mutations are associated with predisposition to breast and ovarian cancer. Nature. 2012 Dec 16;493(7432):406–10.

3. Helleday T, Eshtad S, Nik-Zainal S. Mechanisms underlying mutational signatures in human cancers. Nature Publishing Group. Nature Publishing Group; 2014 Jul 1;15(9):585–98.

4. Li H, Durbin R. Fast and accurate short read alignment with Burrows-Wheeler transform. Bioinformatics. 2009 Jul 15;25(14):1754–60.

5. McKenna A, Hanna M, Banks E, Sivachenko A, Cibulskis K, Kernytsky A, et al. The Genome Analysis Toolkit: a MapReduce framework for analyzing next-generation DNA sequencing data. Genome Res. Cold Spring Harbor Lab; 2010 Sep;20(9):1297–303.

6. Wang K, Li M, Hakonarson H. ANNOVAR: functional annotation of genetic variants from high-throughput sequencing data. Nucleic Acids Research. 2010 Sep;38(16):e164–4.

7. Kircher M, Witten DM, Jain P, O'Roak BJ, Cooper GM, Shendure J. A general framework for estimating the relative pathogenicity of human genetic variants. Nature Publishing Group. Nature Publishing Group; 2014 Mar;46(3):310–5.

8. Price AL, Patterson NJ, Plenge RM, Weinblatt ME, Shadick NA, Reich D. Principal components analysis corrects for stratification in genome-wide association studies. Nat Genet. 2006 Jul 23;38(8):904–9.

9. Desmet F-O, Hamroun D, Lalande M, Collod-Béroud G, Claustres M, Béroud C. Human Splicing Finder: an online bioinformatics tool to predict splicing signals. Nucleic Acids Research. 2009 May;37(9):e67–7.

10. Yeo G, Burge CB. Maximum entropy modeling of short sequence motifs with applications to RNA splicing signals. J Comput Biol. Mary Ann Liebert, Inc; 2004;11(2-3):377–94.

11. Roberts NJ, Norris AL, Petersen GM, Bondy ML, Brand R, Gallinger S, et al. Whole Genome Sequencing Defines the Genetic Heterogeneity of Familial Pancreatic Cancer. Cancer Discovery. 2016 Feb 4;6(2):166–75.
