# Supplementary figures and images for: A region-based gene association study combined with a leave-one-out sensitivity analysis identifies SMG1 as a pancreatic cancer susceptibility gene
Source: PLoS Genet. 2019 Aug 30;15(8):e1008344. doi: 10.1371/journal.pgen.1008344 (PMC6742418; doi:10.1371/journal.pgen.1008344)

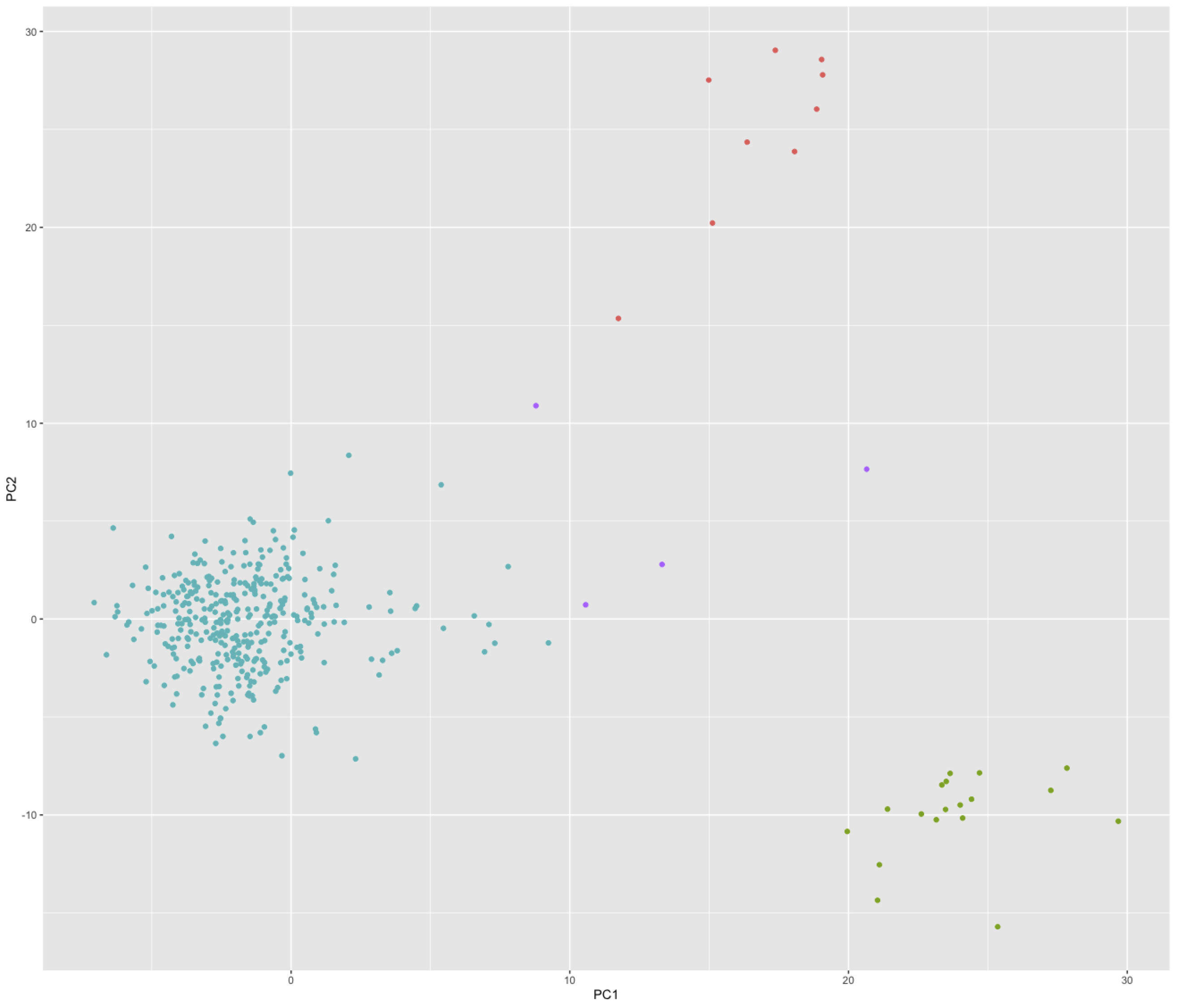

Supplement: S1 Fig — Exon and splicing variants with a MAF >5% in 710 cancer-related genes. Blue dots represent individuals that clustered in the European ancestry group, green dots represent individuals that clustered in the South/Central American ancestry group, and red dots represent individuals that clustered in the Asian ancestry group. Individuals of mixed ancestry indicated by the purple dots were removed from further analyses. (TIFF) [file pgen.1008344.s004.tiff]

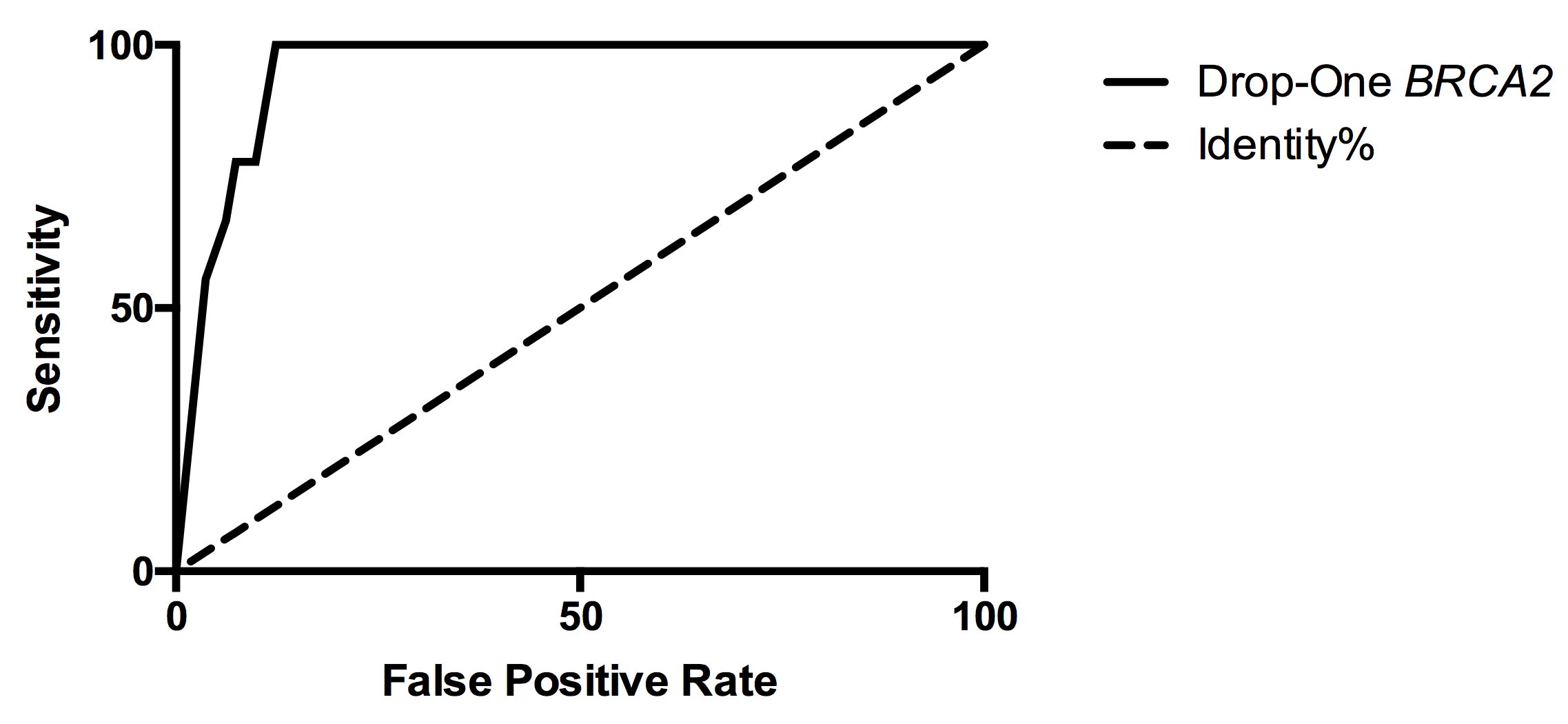

Supplement: S2 Fig — Solid line represents the sensitivity and false positive rate for different p-value increase thresholds (5%-105%) for the LOO-V analysis for BRCA2. The dotted line represents the identity line for a 50/50 test. (TIFF) [file pgen.1008344.s005.tiff]
