# Supplementary material for: A region-based gene association study combined with a leave-one-out sensitivity analysis identifies SMG1 as a pancreatic cancer susceptibility gene
Source: PLoS Genet. 2019 Aug 30;15(8):e1008344. doi: 10.1371/journal.pgen.1008344 (PMC6742418; doi:10.1371/journal.pgen.1008344)
Supplement: S1 Table — (DOCX) [file pgen.1008344.s006.docx]

**Supplemental Table 1. List of 710 cancer-related genes sequenced in Series B of the discovery series.**

| **Gene** |
| --- |
| AATF |
| ABCB11 |
| ABL1 |
| ACSL3 |
| AGAP1 |
| AHR |
| AKT1 |
| ALDH2 |
| ALKBH1 |
| ALKBH2 |
| ALKBH3 |
| ANKLE1 |
| AP1B1 |
| AP2B1 |
| AP3B2 |
| APC |
| APEX1 |
| APEX2 |
| APITD1 |
| APLF |
| APTX |
| AR |
| AREG |
| ARHGAP26 |
| ARHGEF12 |
| ARID1A |
| ARID2 |
| ARIH1 |
| ASF1A |
| ASTE1 |
| ASXL1 |
| ATF2 |
| ATM |
| ATMIN |
| ATP1A1 |
| ATP2B3 |
| ATR |
| ATRIP |
| ATRX |
| ATXN3 |
| AURKA |
| AXIN1 |
| AXIN2 |
| BABAM1 |
| BAP1 |
| BARD1 |
| BAX |
| BAZ1B |
| BCCIP |
| BCL10 |
| BCL11B |
| BCL7A |
| BCOR |
| BLM |
| BMPR1A |
| BRAP |
| BRCA1 |
| BRCA2 |
| BRD3 |
| BRE |
| BRIP1 |
| BTG1 |
| BTG2 |
| BUB1 |
| BUB1B |
| C11orf30 |
| C15orf42 (TICRR) |
| C17orf70 |
| C19orf40 |
| C1orf124 (SPRTN) |
| C9orf102 (ERCC6L2) |
| C9orf80 (INIP) |
| CALR |
| CAMTA1 |
| CAND1 |
| CARS |
| CASC5 |
| CASP3 |
| CASP8 |
| CAV1 |
| CBFA2T3 |
| CCDC6 |
| CCNA1 |
| CCNA2 |
| CCNB1 |
| CCNB1IP1 |
| CCND1 |
| CCNE1 |
| CCNH |
| CCNO |
| CD79A |
| CDC14B |
| CDC25A |
| CDC25B |
| CDC25C |
| CDC45 |
| CDC6 |
| CDC73 |
| CDH1 |
| CDH11 |
| CDH13 |
| CDK1 |
| CDK12 |
| CDK2 |
| CDK4 |
| CDK6 |
| CDK7 |
| CDKN1B |
| CDKN2A |
| CDKN2C |
| CDKN2D |
| CDS2 |
| CEBPA |
| CEBPG |
| CEP164 |
| CETN2 |
| CFTR |
| CHAF1A |
| CHAF1B |
| CHD1L |
| CHD4 |
| CHEK1 |
| CHEK2 |
| CHIC2 |
| CHRNA4 |
| CIB1 |
| CIC |
| CIITA |
| CINP |
| CLP1 |
| CLSPN |
| CLTCL1 |
| CNOT3 |
| CNTLN |
| CNTRL |
| COBRA1 |
| COL1A1 |
| COL7A1 |
| CPA1 |
| CRB2 |
| CREB1 |
| CREB3L1 |
| CREBBP |
| CRY1 |
| CRY2 |
| CSNK1D |
| CSNK1E |
| CSTF1 |
| CSTF2 |
| CTBP1 |
| CTCFL |
| CTRC |
| CUL4A |
| CUL4B |
| CYLD |
| CYP19A1 |
| CYP1A1 |
| DAPK1 |
| DAXX |
| DBF4 |
| DCLRE1A |
| DCLRE1B |
| DCLRE1C |
| DDB1 |
| DDB2 |
| DDR1 |
| DDX1 |
| DDX19B |
| DDX5 |
| DHX9 |
| DICER1 |
| DIS3L2 |
| DKC1 |
| DMC1 |
| DNA2 |
| DNM2 |
| DOCK8 |
| DOT1L |
| DTL |
| DUSP3 |
| DYRK2 |
| E2F1 |
| E2F2 |
| E2F4 |
| E2F6 |
| EBF1 |
| ECEL1 |
| EEPD1 |
| EGFR |
| EIF4A2 |
| EIF4EBP1 |
| ELANE |
| EME1 |
| EME2 |
| EP300 |
| EPC2 |
| EPCAM |
| ERBB2 |
| ERCC1 |
| ERCC2 |
| ERCC3 |
| ERCC4 |
| ERCC5 |
| ERCC6 |
| ERCC8 |
| ESCO1 |
| ESCO2 |
| ESR1 |
| ETS1 |
| ETS2 |
| ETV7 |
| EXO1 |
| EXT1 |
| EXT2 |
| EYA1 |
| EYA2 |
| EYA3 |
| EYA4 |
| FAH |
| FAM175A |
| FAM46C |
| FAN1 |
| FANCA |
| FANCB |
| FANCC |
| FANCD2 |
| FANCE |
| FANCF |
| FANCG |
| FANCI |
| FANCL |
| FANCM |
| FAS |
| FBXO11 |
| FBXO18 |
| FBXO6 |
| FBXW7 |
| FEN1 |
| FGF10 |
| FGFR2 |
| FH |
| FHIT |
| FHL2 |
| FLCN |
| FOS |
| FOXM1 |
| FOXO3 |
| FOXP1 |
| FTO |
| FUBP1 |
| FZR1 |
| GADD45A |
| GADD45G |
| GATA1 |
| GATA3 |
| GBA |
| GEN1 |
| GJB2 |
| GPC3 |
| GSTCD |
| GSTP1 |
| GTF2H1 |
| GTF2H2C |
| GTF2H3 |
| GTF2H4 |
| GTF2H5 |
| H2AFX |
| HDAC1 |
| HDAC2 |
| HERPUD1 |
| HFE |
| HIC1 |
| HINFP |
| HMBS |
| HMG20B |
| HMGB1 |
| HMGB2 |
| HNF1A |
| HOOK3 |
| HOXA11 |
| HOXA9 |
| HSP90AB1 |
| HSPA5 |
| HUS1 |
| ID4 |
| IFI16 |
| IFNB1 |
| IGF1 |
| IGHMBP2 |
| IKBKG |
| IKZF1 |
| IL2 |
| INO80 |
| INTS3 |
| IRS1 |
| ITGA6 |
| ITIH2 |
| ITK |
| JAK1 |
| JAK2 |
| JMY |
| JUN |
| KAT2B |
| KAT5 |
| KAT6B |
| KCNH6 |
| KDM5A |
| KDM5C |
| KDM6A |
| KDSR |
| KIF22 |
| KIN |
| KLF6 |
| KLK3 |
| KPNA2 |
| KRAS |
| KRT5 |
| LIG1 |
| LIG3 |
| LIG4 |
| LMO4 |
| LMO7 |
| LRIG3 |
| LTB |
| MAP2K4 |
| MAP3K1 |
| MAX |
| MBD2 |
| MBD3 |
| MBD4 |
| MC1R |
| MCPH1 |
| MDC1 |
| MDM2 |
| MDM4 |
| MDS2 |
| MED17 |
| MED21 |
| MEN1 |
| MGMT |
| MLH1 |
| MLH3 |
| MMS19 |
| MN1 |
| MNAT1 |
| MNX1 |
| MORF4L1 |
| MORF4L2 |
| MPG |
| MRE11A |
| MSH2 |
| MSH3 |
| MSH4 |
| MSH5 |
| MSH6 |
| MTAP |
| MUM1 |
| MUS81 |
| MUTYH |
| MYC |
| MYH11 |
| NBN |
| NBR1 |
| NCOA2 |
| NCOA3 |
| NCOA6 |
| NDRG1 |
| NEIL1 |
| NEIL2 |
| NEIL3 |
| NEK1 |
| NEK11 |
| NF1 |
| NF2 |
| NFKB1 |
| NHEJ1 |
| NIN |
| NINL |
| NME1 |
| NONO |
| NOTCH1 |
| NPM1 |
| NR1H2 |
| NR2E3 |
| NR4A3 |
| NSMCE1 |
| NSMCE2 |
| NTHL1 |
| NUDT1 |
| NUFIP1 |
| NUMA1 |
| NUP98 |
| OBFC2A |
| OBFC2B |
| OGG1 |
| OPTC |
| PAFAH1B2 |
| PALB2 |
| PARG |
| PARP1 |
| PARP2 |
| PARP3 |
| PARP4 |
| PATZ1 |
| PAX5 |
| PAX7 |
| PBRM1 |
| PCM1 |
| PCNA |
| PDCD1LG2 |
| PDLIM4 |
| PER1 |
| PGM3 |
| PGR |
| PHF6 |
| PHOX2B |
| PIK3R1 |
| PLAT |
| PLK1 |
| PLK3 |
| PML |
| PMS1 |
| PMS2 |
| PNKP |
| POLA1 |
| POLB |
| POLD1 |
| POLD2 |
| POLD3 |
| POLD4 |
| POLE |
| POLE2 |
| POLG |
| POLG2 |
| POLH |
| POLI |
| POLK |
| POLL |
| POLN |
| POLQ |
| POLR2A |
| POLR2H |
| POLR2K |
| POT1 |
| POU2F1 |
| POU4F1 |
| POU4F2 |
| PPM1D |
| PPP1CA |
| PPP2R5A |
| PPP2R5B |
| PPP2R5C |
| PPP2R5D |
| PPP2R5E |
| PPP4C |
| PRDM1 |
| PRF1 |
| PRKAR1A |
| PRKCG |
| PRKDC |
| PRMT6 |
| PRPF19 |
| PRSS1 |
| PSMD3 |
| PTCH1 |
| PTEN |
| PTPRC |
| PTPRH |
| PTTG1 |
| RAD1 |
| RAD17 |
| RAD18 |
| RAD21 |
| RAD23A |
| RAD23B |
| RAD50 |
| RAD51 |
| RAD51AP1 |
| RAD51B |
| RAD51C |
| RAD51D |
| RAD52 |
| RAD54B |
| RAD54L |
| RAD54L2 |
| RAD9A |
| RAD9B |
| RALGDS |
| RANBP17 |
| RASGRF1 |
| RASSF1 |
| RB1 |
| RBBP4 |
| RBBP7 |
| RBBP8 |
| RBL1 |
| RBL2 |
| RBM14 |
| RBX1 |
| RDM1 |
| RECQL |
| RECQL4 |
| RECQL5 |
| RELA |
| REV1 |
| REV3L |
| RFC1 |
| RFC2 |
| RFC3 |
| RFC4 |
| RFC5 |
| RFWD2 |
| RFWD3 |
| RHNO1 |
| RHOH |
| RINT1 |
| RMI2 |
| RNF11 |
| RNF144B |
| RNF168 |
| RNF43 |
| RNF8 |
| RPA1 |
| RPA2 |
| RPA3 |
| RPA4 |
| RPAIN |
| RPL10 |
| RPL22 |
| RPL5 |
| RPS27L |
| RPS3 |
| RRAD |
| RRM2B |
| RTEL1 |
| RUNX1 |
| RUVBL2 |
| SBDS |
| SDC4 |
| SDHA |
| SDHAF2 |
| SDHB |
| SDHC |
| SDHD |
| SERPINA1 |
| SETD2 |
| SETMAR |
| SETX |
| SFPQ |
| SH2B3 |
| SH2D1A |
| SHFM1 |
| SHPRH |
| SIRT1 |
| SLC25A13 |
| SLC30A9 |
| SLC45A3 |
| SLK |
| SLX1A |
| SLX4 |
| SMAD3 |
| SMAD4 |
| SMARCA1 |
| SMARCA2 |
| SMARCA4 |
| SMARCB1 |
| SMARCD2 |
| SMARCE1 |
| SMC1A |
| SMC3 |
| SMC5 |
| SMC6 |
| SMG1 |
| SMUG1 |
| SMURF2 |
| SOCS1 |
| SOD1 |
| SP1 |
| SPO11 |
| SPP1 |
| SRBD1 |
| SRGAP3 |
| SRY |
| SSRP1 |
| STAG2 |
| STAT1 |
| STAT3 |
| STAT5A |
| STK11 |
| STRA13 |
| SUCLA2 |
| SUFU |
| SUGT1 |
| SUMO1 |
| SUPT16H |
| SUPT6H |
| SUZ12 |
| SYK |
| TAOK1 |
| TAOK2 |
| TAOK3 |
| TAPBP |
| TCF3 |
| TCF7L2 |
| TCHP |
| TDG |
| TDP1 |
| TELO2 |
| TERF1 |
| TERF2 |
| TERF2IP |
| TERT |
| TET1 |
| TET2 |
| TFDP1 |
| TFDP2 |
| TFE3 |
| TFEB |
| TGFBR1 |
| THRAP3 |
| TMEM127 |
| TMEM161A |
| TMPRSS2 |
| TMPRSS7 |
| TNFAIP3 |
| TNFRSF14 |
| TNP1 |
| TOP1 |
| TOP2A |
| TOP3A |
| TOPBP1 |
| TOX3 |
| TP53 |
| TP53BP1 |
| TP73 |
| TRAF7 |
| TREX1 |
| TREX2 |
| TRIM24 |
| TRIM37 |
| TRIM40 |
| TRIP11 |
| TRIP13 |
| TSC1 |
| TSC2 |
| TSHR |
| TSPAN17 |
| TTC13 |
| TTC5 |
| TTL |
| TUBG1 |
| TUBGCP4 |
| TUBGCP5 |
| TUBGCP6 |
| TYMS |
| UBA1 |
| UBE2A |
| UBE2B |
| UBE2D1 |
| UBE2D3 |
| UBE2I |
| UBE2L3 |
| UBE2N |
| UBE2V1 |
| UBE2V2 |
| UBE4A |
| UBE4B |
| UBR5 |
| UCP2 |
| UHRF1 |
| UIMC1 |
| UNG |
| UPF1 |
| UROD |
| USP1 |
| USP28 |
| USP3 |
| UVRAG |
| VCP |
| VEGFA |
| VHL |
| WAS |
| WDR16 |
| WDR33 |
| WEE1 |
| WIF1 |
| WRN |
| WRNIP1 |
| WT1 |
| WWP1 |
| WWP2 |
| XAB2 |
| XPA |
| XPC |
| XRCC1 |
| XRCC2 |
| XRCC3 |
| XRCC4 |
| XRCC5 |
| XRCC6 |
| XRCC6BP1 |
| YWHAH |
| YY1 |
| ZBTB16 |
| ZMYM2 |
| ZNF331 |
| ZNF350 |
| ZNF384 |
| ZRSR2 |
| ZSWIM7 |
