# Supplementary material for: A region-based gene association study combined with a leave-one-out sensitivity analysis identifies SMG1 as a pancreatic cancer susceptibility gene
Source: PLoS Genet. 2019 Aug 30;15(8):e1008344. doi: 10.1371/journal.pgen.1008344 (PMC6742418; doi:10.1371/journal.pgen.1008344)
Supplement: S2 Table — The majority of variants identified in these 4 candidate genes were identified in the control series and were thus excluded from further analyses. MAF for our case series, control series, and 3 public databases as well as the CADD score are shown. The p-value increase observed for the leave-one-out variant test is indicated. CADD, combined annotation depletion dependent. MAF, minor allele frequency. EVS, Exome. Variant Server. ExAC, Exome Aggregation Consortium. 1000s, 1000 genomes project. (DOCX) [file pgen.1008344.s007.docx]

**Supplemental Table 2. Summary of variants identified in the discovery series by the leave-one-out analysis for 4 genes with a significant association in MiST.**

| Gene | Mutation | Type | CADD | Case MAF | Control MAF | EVS | ExAC | 1000s | p-value increase (%) |
| --- | --- | --- | --- | --- | --- | --- | --- | --- | --- |
| *STK11* | c.316C>T:p.R106W | Missense | 33 | 0 | 0.0010 | . | 1.65E-5 | . | >45% |
| *STK11* | c.1062C>G:p.F354L | Missense | 0.838 | 0.0013 | 0.0051 | 0.0038 | 0.0049 | . | >105% |
| *CHEK2* | c.1556G>T:p.R519L | Missense | 24.9 | 0.0013 | 0 | . | 2.18E-4 | . | >45% |
| *CHEK2* | c.1525C>T:p.P509S | Missense | 8.9 | 0 | 0.0081 | . | 1.13E-4 | . | >105% |
| *CHEK2* | c.1427C>T:p.T476M | Missense | 31 | 0.0013 | 0 | 4.01E-4 | 3.7E-4 | . | >75% |
| *CHEK2* | c.1312G>T:p.D438Y | Missense | 33 | 0 | 0.0020 | 3.08E-4 | 2.80E-4 | . | >105% |
| *CHEK2* | c.1283C>T:p.S428F | Missense | 29.8 | 0.0026 | 0 | 1.54E-4 | 3.05E-4 | . | >105% |
| *CHEK2* | c.1100delC:p.T367fs | Frameshift | 35 | 0 | 0.0030 | . | . | . | >35% |
| *CHEK2* | c.470T>C:p.I157T | Missense | 21.1 | 0.0013 | 0 | 0.0016 | 0.0041 | . | >55% |
| *CHEK2* | c.254C>T:p.P85L | Missense | 17.3 | 0.0039 | 0 | 0.0024 | 8.81E-4 | . | >105% |
| *CHEK2* | c.7C>T:p.R3W | Missense | 34 | 0.0013 | 0 | 1.54E-4 | 2.31E-4 | . | >75% |
| *RECQL* | c.1460A>C:p.K487T | Missense | 14.4 | 0.0104 | 0.0005 | 0.017 | 0.0057 | . | >105% |
| *RECQL* | c.304G>A:p.V102I | Missense | 21.1 | 0 | 0.0056 | 0.048 | 0.013 | . | >105% |
| *RECQL* | c.207T>A:p.N69K | Missense | 11.8 | 0 | 0.0061 | . | 9.06E-5 | . | >105% |
| *TDG* | c.56C>T:p.T19M | Missense | 16.9 | 0.0026 | 0 | 2.31E-4 | 5.52E-4 | . | >105% |
| *TDG* | c.235C>A:p.P79T | Missense | 16.0 | 0.0026 | 0 | . | 0.0053 | . | >105% |
| *TDG* | c.410T>C:p.I137T | Missense | 26.9 | 0.0013 | 0 | . | 8.24E-6 | . | >105% |
| *TDG* | c.547G>A:p.G183R | Missense | 17.7 | 0.0013 | 0 | . | 8.24E-6 | . | >105% |
| *TDG* | c.602A>C:p.K201T | Missense | 22.2 | 0.0013 | 0 | . | 0.029 | . | >105% |
| *TDG* | c.964+2delTAAGG | Splicing | 14.3 | 0 | 0.0015 | . | . | . | >45% |
| *TDG* | c.964+2T>G | Splicing | 25.6 | 0 | 0.0081 | . | 1.65E-5 | . | >105% |
| *TDG* | c.1090insTTGAGAGC | Frameshift | 34.0 | 0 | 0.011 | . | . | . | >55% |

The majority of variants identified in these 4 candidate genes were identified in the control series and were thus excluded from further analyses. MAF for our case series, control series, and 3 public databases as well as the CADD score are shown. The p-value increase observed for the leave-one-out variant test is indicated. CADD, combined annotation depletion dependent. MAF, minor allele frequency. EVS, Exome. Variant Server. ExAC, Exome Aggregation Consortium. 1000s, 1000 genomes project.
