# Supplementary material for: A region-based gene association study combined with a leave-one-out sensitivity analysis identifies SMG1 as a pancreatic cancer susceptibility gene
Source: PLoS Genet. 2019 Aug 30;15(8):e1008344. doi: 10.1371/journal.pgen.1008344 (PMC6742418; doi:10.1371/journal.pgen.1008344)
Supplement: S3 Table — The CADD score and minor allele frequency (MAF) for each variant in the case series, in the controls and in the 3 public databases are shown. CADD, combined annotation depletion dependent. EVS, Exome. Variant Server. ExAC, Exome Aggregation Consortium. 1000s, 1000 genomes project. LOO-V, leave-one-out variant analysis. (DOCX) [file pgen.1008344.s008.docx]

**Supplemental Table 3. *SMG1* variants identified in the validation series.**

| Gene | Variant | Type | CADD | Cases MAF | Controls MAF | EVS | ExAC | 1000s | Identified in Discovery Series  (# cases / controls) | Identified in LOO-V in Discovery Series |
| --- | --- | --- | --- | --- | --- | --- | --- | --- | --- | --- |
| *SMG1* | c.103G>A:p.A35T | Missense | 21.8 | 0.01 | 0.0027 | 0.0027 | 0.019 | . | 7/1 | Yes |
| *SMG1* | c.202C>T:p.R68W | Missense | 25.7 | 0.0019 | 6.6E-4 | 8.6E-5 | 9.1E-5 | . | . | . |
| *SMG1* | c.1158A>C:p.E386D | Missense | 23.2 | . | 6.6E-4 | . | . | . | . | . |
| *SMG1* | c.1756C>A:p.P586T | Missense | 6.595 | . | 6.6E-4 | . | . | . | . | . |
| *SMG1* | c.2014T>G:p.C672G | Missense | 25.5 | . | 6.6E-4 | . | 5.3E-5 | . | . | . |
| *SMG1* | c.2185A>G:p.K729E | Missense | 13.21 | 9.4E-4 | . | . | . | . | . | . |
| *SMG1* | c.2531C>T:p.A844V | Missense | 27.8 | . | 6.6E-4 | . | 8.6E-6 | . | . | . |
| *SMG1* | c.2597T>C:p.I866T | Missense | 26.1 | 9.4E-4 | . | . | 8.5E-6 | . | . | . |
| *SMG1* | c.2711A>G:p.N904S | Missense | 9.07 | 9.4E-4 | . | . | 1.2E-4 | . | . | . |
| *SMG1* | c.3509C>G:p.T1170S | Missense | 15.12 | . | 6.6E-4 | . | 1.3E-4 | . | . | . |
| *SMG1* | c.3524C>T:p.P1175L | Missense | 22.9 | 9.4E-4 | 6.6E-4 | 1.7E-4 | 3.9E-4 |  | . | . |
| *SMG1* | c.3647G>T:p.S1216I | Missense | 20.7 | . | 6.6E-4 | . | . | . | . | . |
| *SMG1* | c.3917C>T:p.P1306L | Missense | 8.947 | 0.0047 | 0.0027 | 0.0018 | 0.0017 | . | 4/1 | Yes |
| *SMG1* | c.4064A>T:p.Y1355F | Missense | 21.2 | 9.4E-4 | . | . | . | . | . | . |
| *SMG1* | c.4085A>C:p.N1362T | Missense | 15.65 | . | 6.6E-4 | . | 1.8E-5 | . | . | . |
| *SMG1* | c.4147C>T:p.R1383C | Missense | 23.5 | . | 6.6E-4 | . | . | . | . | . |
| *SMG1* | c.4237C>A:p.Q1413K | Missense | 11.7 | . | 6.6E-4 | . | . | . | . | . |
| *SMG1* | c.4501A>G:p.M1501V | Missense | 23.4 | . | 6.6E-4 | . | 1.7E-5 | . | . | . |
| *SMG1* | c.4670G>A:p.G1557D | Missense | 17.05 | 0.0019 | 6.6E-4 | 4.2E.4 | 6.0E-4 | . | . | . |
| *SMG1* | c.5696G>A:p.C1899Y | Missense | 23.1 | 9.4E-4 | . | . | 4.1E-4 | . | . | . |
| *SMG1* | c.5842-1G>T | Splicing | 28.3 | 9.4E-4 | . | . | . | . | . | . |
| *SMG1* | c.5984A>C:p.N1995T | Missense | 23.1 | 0.0019 | 6.6E-4 | 1.6E-4 | 2.7E-4 | . | . | . |
| *SMG1* | c.6127A>G:p.N2043D | Missense | 22.5 | . | 6.6E-4 | 1.7E-4 | 8.3E-6 | . | . | . |
| *SMG1* | c.6265A>G:p.I2089V | Missense | 5.952 | . | 6.6E-4 | . | . | . | . | . |
| *SMG1* | c.6355G>A:p.V2119I | Missense | 22.9 | 9.4E-4 | . | . | . | . | . | . |
| *SMG1* | c.7559A>G:p.E2520G | Missense | 24.6 | 9.4E-4 | . | . | 2.5E-5 | . | . | . |
| *SMG1* | c.7615C>T:p.H2539Y | Missense | 22.5 | 9.4E-4 | . | . | . | . | . | . |
| *SMG1* | c.7729A>G:p.T2577A | Missense | 11.96 | 9.4E-4 | . | . | 8.3E-6 | . | . | . |
| *SMG1* | c.7804G>A:p.A2602T | Missense | 22.6 | 9.4E-4 | . | 7.1E-4 | 1.7E-5 | . | 0/2 | . |
| *SMG1* | c.7839T>G:p.I2613M | Missense | 23.4 | . | 6.6E-4 | . | 5.8E-5 | . | . | . |
| *SMG1* | c.7874C>A:p.A2625D | Missense | 15.12 | 9.4E-4 | . | . | . | . | . | . |
| *SMG1* | c.8476C>T:p.H2826Y | Missense | 22.6 | 9.4E-4 | . | 5.7E-4 | 2.1E-4 | . | . | . |
| *SMG1* | c.8482G>T:p.V2828L | Missense | 22.7 | . | 6.6E-4 | 1.6E-4 | 1.0E-4 | . | 1/2 | . |
| *SMG1* | c.8782G>A:p.D2928N | Missense | 22.5 | . | 6.6E-4 | . | 8.3E-6 | . | . | . |
| *SMG1* | c.8842G>T:p.V2948F | Missense | 24 | 0.0019 | . | 8.3E-5 | 2.5E-4 | . | 1/0 | . |
| *SMG1* | c.8845G>A:p.D2949N | Missense | 18.47 | . | 6.6E-4 | 8.3E-5 | 1.1E-4 | . | . | . |
| *SMG1* | c.9140G>A:p.S3047N | Missense | 15.81 | . | 6.6E-4 | 8.5E-5 | 1.2E-4 | . | . | . |
| *SMG1* | c.9182T>C:p.I3061T | Missense | 23 | 9.4E-4 | . | . | 8.3E-6 | . | . | . |
| *SMG1* | c.9839C>G:p.A3280G | Missense | 22.4 | 9.4E-4 | . | . | 1.7E-5 | . | . | . |
| *SMG1* | c.9884G>C:p.R3295T | Missense | 26.9 | 9.4E-4 | . | 1.7E-4 | 1.7E-5 | . | . | . |
| *SMG1* | c.10401A>T:p.Q3467H | Missense | 25.4 | . | 6.6E-4 | . | . | . | Yes – 1/0 | . |
| *SMG1* | c.10814G>A:p.R3605K | Missense | 26.6 | . | 6.6E-4 | . | . | . | . | . |
| *SMG1* | c.10897G>A:p.V3633I | Missense | 25.2 | . | 6.6E-4 | . | . | . | . | . |

The CADD score and minor allele frequency (MAF) for each variant in the case series, in the controls and in the 3 public databases are shown. CADD, combined annotation depletion dependent. EVS, Exome. Variant Server. ExAC, Exome Aggregation Consortium. 1000s, 1000 genomes project. LOO-V, leave-one-out variant analysis.
